# Extensive mucosal denudation and necrosis complicated by esophagothoracic fistula after submucosal tunnel endoscopic resection

**DOI:** 10.1055/a-2638-5696

**Published:** 2025-07-10

**Authors:** Lixing Yu, Jiatao Tu, Xuan Huang

**Affiliations:** 1 74723 Department of Gastroenterology, The First Affiliated Hospital of Zhejiang Chinese Medical University, Hangzhou, China

A 21-year-old woman with no underlying medical history was admitted for an esophageal mass. After excluding contraindications, submucosal tunnel endoscopic resection (STER) was performed ( Fig. 1 ).

**Fig. 1:**
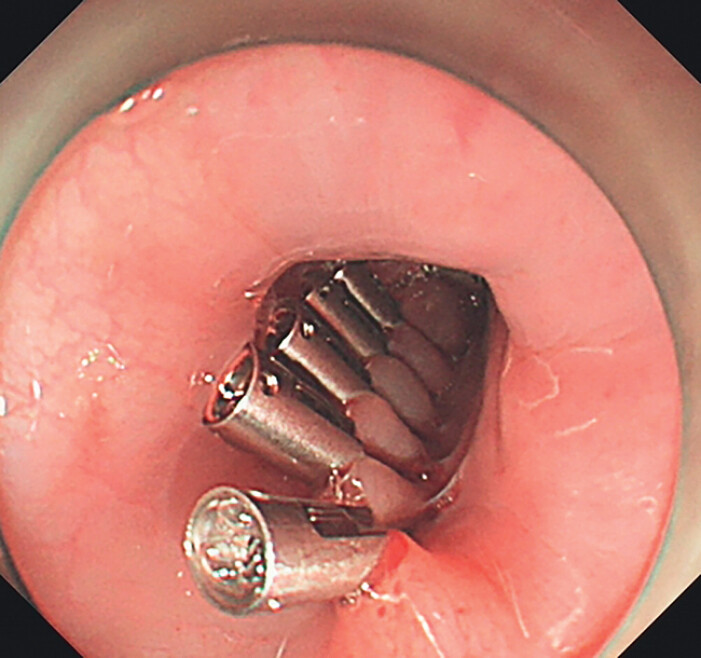
The wound bed post-submucosal tunnel endoscopic resection was securely closed using hemostatic clips.

On postoperative day 1, the patient developed a fever, chest tightness, shortness of breath, and chest pain. Contrast esophagography demonstrated contrast extravasation, indicative of an esophagothoracic fistula ( Fig. 2 ). A left-sided tube thoracostomy was performed for closed drainage of the thoracic cavity, with two chest tubes placed in total, and a large amount of purulent fluid was drained.

**Fig. 2:**
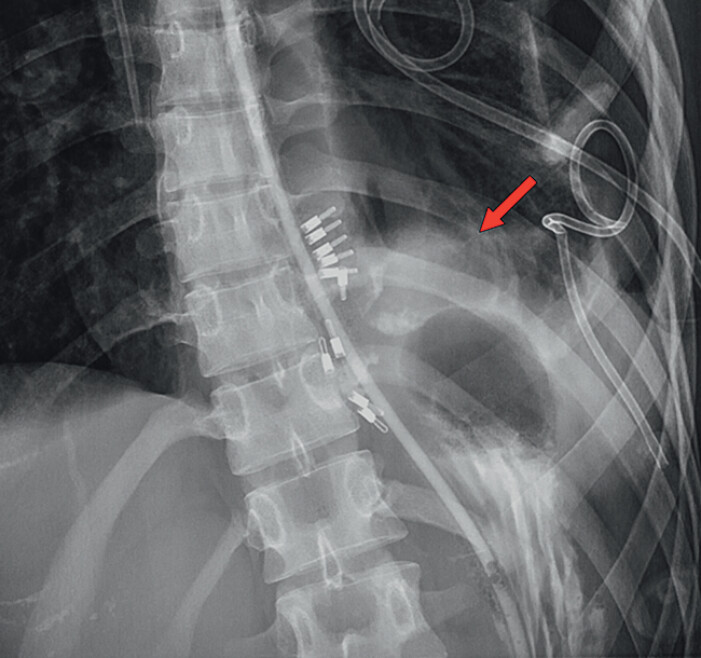
Contrast esophagography demonstrated peri-procedural contrast extravasation, indicative of leakage at the surgical site.

Subsequent endoscopic evaluation revealed extensive and confluent mucosal denudation with necrosis extending from 35 to 40 cm distal to the incisors, accompanied by necrotic ulceration at the gastroesophageal junction ( Fig. 3 **a–c** ); the retained hemostatic clips were carefully removed under direct visualization, followed by successful closure of the suspected fistula orifice ( Fig. 3 **d** , Video 1 ).

**Fig. 3:**
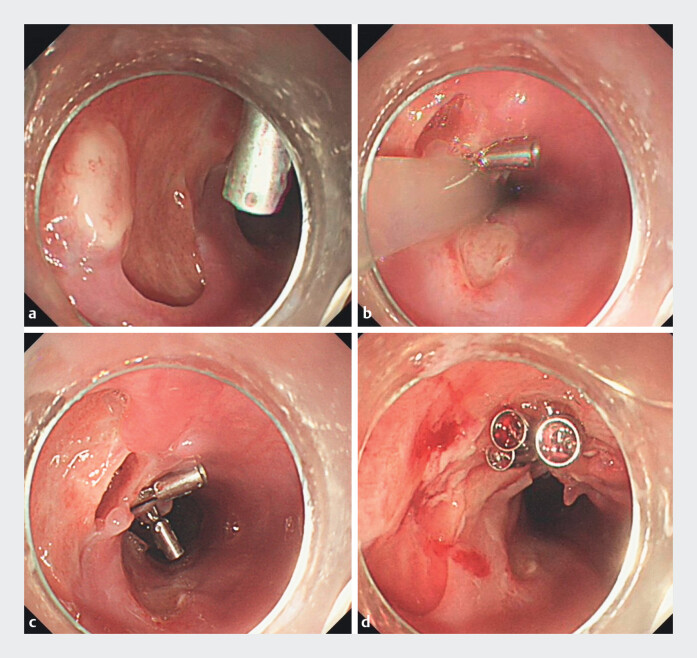
**a–c** Endoscopy showed extensive and confluent mucosal denudation with necrosis. **d** Endoscopic clip closure was performed on the suspected fistula orifice.

Extensive postoperative mucosal necrosis complicated by esophagothoracic fistula successfully managed through a combination of endoscopic intervention and enhanced nutritional support therapy.Video 1

Following endoscopic intervention, antimicrobial therapy combined with enteral and parenteral nutritional support was administered, with no significant adverse events or discomfort observed during the treatment course. Contrast esophagography on postoperative day 7 confirmed the absence of contrast medium leakage into the thoracic cavity. A three-month follow-up endoscopy demonstrated satisfactory mucosal healing at the previous surgical site ( Fig. 4 ).

**Fig. 4:**
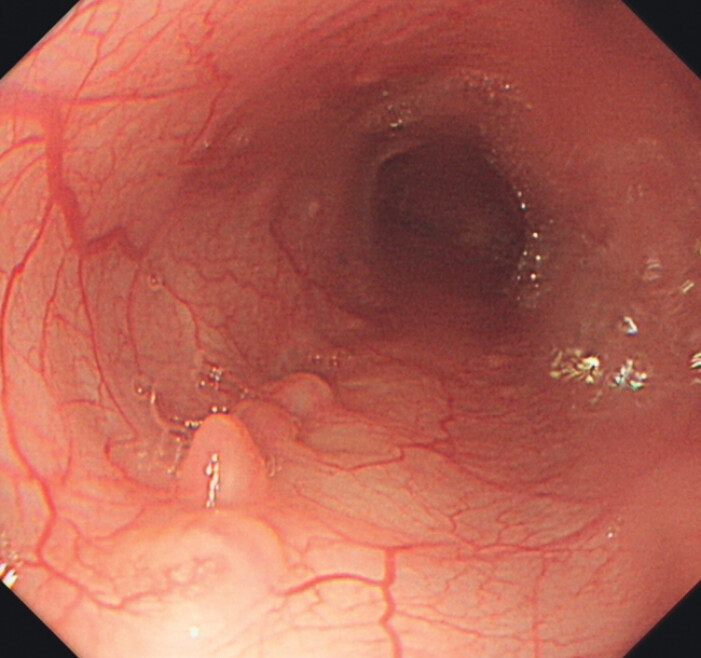
Endoscopic evaluation revealed satisfactory mucosal healing of the esophagus at three months postoperatively.

Extensive esophageal mucosal necrosis with esophagothoracic fistula represents a rare complication following STER 1 . Postoperative analysis suggested potential contributing factors including extensive submucosal vascular dissection during the procedure, inadequate closure of the tunnel entrance, and suboptimal postoperative nutritional support. The patient ultimately achieved complete recovery through comprehensive endoscopic management combined with adjunctive therapies. These findings indicate that extensive postoperative mucosal necrosis complicated by esophagothoracic fistula could be managed through a combination of endoscopic intervention and enhanced nutritional support therapy.

Endoscopy_UCTN_Code_CPL_1AH_2AG

## Notes

Endoscopy E-Videos https://eref.thieme.de/e-videos
*E-Videos* is an open access online section of the journal *Endoscopy* , reporting on interesting cases and new techniques in gastroenterological endoscopy. All papers include a high-quality video and are published with a Creative Commons CC-BY license. Endoscopy E-Videos qualify for HINARI discounts and waivers and eligibility is automatically checked during the submission process. We grant 100% waivers to articles whose corresponding authors are based in Group A countries and 50% waivers to those who are based in Group B countries as classified by Research4Life (see: https://www.research4life.org/access/eligibility/ ). This section has its own submission website at https://mc.manuscriptcentral.com/e-videos .
